# Gradient Microstructure Induced by Surface Mechanical Attrition Treatment (SMAT) in Magnesium Studied Using Positron Annihilation Spectroscopy and Complementary Methods

**DOI:** 10.3390/ma13184002

**Published:** 2020-09-09

**Authors:** Konrad Skowron, Ewa Dryzek, Mirosław Wróbel, Paweł Nowak, Marianna Marciszko-Wiąckowska, Léa Le Joncour, Manuel François, Benoit Panicaud, Andrzej Baczmański

**Affiliations:** 1 Institute of Nuclear Physics, Polish Academy of Sciences, PL-31342 Kraków, Poland; ewa.dryzek@ifj.edu.pl; 2 Faculty of Metals Engineering and Industrial Computer Science, AGH University of Science and Technology, al. A. Mickiewicza 30, 30-059 Kraków, Poland; mwrobel@agh.edu.pl; 3 Jerzy Haber Institute of Catalysis and Surface Chemistry, Polish Academy of Sciences, Niezapominajek 8, PL-30239 Kraków, Poland; ncnowak@cyf-kr.edu.pl; 4 Academic Centre for Materials and Nanotechnology, AGH University of Science and Technology, al. A. Mickiewicza 30, 30-059 Kraków, Poland; marciszk@agh.edu.pl; 5 Université de Technologie de Troyes (UTT), LASMIS, 12 Rue Marie Curie, 10010 Troyes, France; lea.le_joncour@utt.fr (L.L.J.); manuel.francois@utt.fr (M.F.); benoit.panicaud@utt.fr (B.P.); 6 Faculty of Physics and Applied Computer Science, AGH University of Science and Technology, al. A. Mickiewicza 30, 30-059 Kraków, Poland; andrzej.baczmanski@fis.agh.edu.pl

**Keywords:** magnesium, surface mechanical attrition treatment, SMAT, gradient structured materials, positron annihilation spectroscopy, corrosion

## Abstract

Surface mechanical attrition treatment (SMAT) was used to generate a gradient microstructure in commercial grade magnesium. Positron annihilation lifetime spectroscopy and variable energy positron beam measurements, as well as microhardness tests, electron backscatter diffraction, X-ray diffraction, and electrochemical corrosion tests, were used to investigate the created subsurface microstructure and its properties. It was found that SMAT causes an increase in dislocation density and grain refinement which results in increased hardness of the subsurface zone. The mean positron lifetime values indicate trapping of positrons in vacancies associated with dislocations and dislocation jogs. The increase of the SMAT duration and the vibration amplitude influences the depth profile of the mean positron lifetime, which reflects the defect concentration profile. Electrochemical measurements revealed that the structure induced by SMAT increases the susceptibility of magnesium to anodic oxidation, leading to the enhanced formation of hydroxide coverage at the surface and, as a consequence, to the decrease in corrosion current. No significant effect of the treatment on the residual stress was found.

## 1. Introduction

In 2019, primary magnesium production worldwide was estimated at 1.1 million tons/year [1]. Global growth in the magnesium market is expected to average 3.4% per year, reaching almost 1.2 million tons/year by 2020 [2]. By far, the largest proportion in magnesium industrial uses is its metallurgical applications, mainly as an alloying element in aluminum, zinc, lead, and other nonferrous alloys or as a reducing agent, oxygen scavenger, and desulfurizer [3]. However, magnesium is also the lightest metal that can be used as a structural material. Its density is significantly lower than that of steel, titanium, and aluminum alloys. It is even lower than the density of most of glass-fiber-reinforced polymers and similar to that of much more expensive carbon–fiber composites [4,5]. Good stiffness and mechanical stability and high thermal and electrical conductivity, as well as good vibration and shock absorption ability, are its properties important in engineering design. However, magnesium is difficult to form at room temperature as a result of an insufficient number of deformation modes (slip systems) due to its hexagonal structure. Mechanical properties of magnesium improve when it is alloyed with a small amount of other metals, hence versatility of cast and wrought magnesium alloys in applications in automotive and aerospace industries. However, in the literature reports, other methods of improving the formability of magnesium without alloying have been proposed [6]. Among other uses, medical applications of magnesium constitute a highly profitable niche market [7]. Magnesium has many advantages compared with traditional metallic biomaterials, i.e., stainless steel, cobalt-chromium based alloys, titanium alloys, etc. Its modulus of elasticity (45 GPa) and density (1.74 g/cm^3^) are close to those of natural bone (20–57 GPa and 1.75–2,1 g/cm^3^, respectively) [8,9]. Compressive and tensile strength of magnesium (20–115 MPa and 90–190 MPa, respectively) are also similar to those of bones (160–240 MPa and 35–283 MPa, respectively) [10]. However, its degradation speed in the body is too fast, and its susceptibility to galvanic corrosion is not acceptable, which was already noticed after its application to leg bone fractures at the end of the 19th century [11,12,13]. Thus, both alloying and different treatments are tried to eliminate these drawbacks, i.e., to decrease its degradation speed and increase its strength [14,15,16,17,18]. Simultaneously, the demand for short-term non-permanent implants has resulted in the development of a new generation of functional resorbable biomaterials, among them, biodegradable magnesium-calcium alloys tested as a potential material for orthopedic implants [19,20,21]. 

Typical magnesium alloys (cast and wrought) contain at least 90% of magnesium, but much smaller amounts of alloying elements can be recommended for some biomedical applications. This limits the potential uses of magnesium strengthening schemes, e.g., grain refinement during solidification by alloying with Al, Si, Sr, Ca, C, or carbon-containing agents [22]. Fortunately, both deformation strengthening and grain refinement as a result of the recrystallization are possible. In magnesium alloys, the typical grain size range usually obtained by static recrystallization is 8–25 μm [23]. Cold hydrostatic extrusion, followed by low-temperature annealing, can produce about 3 μm grains [24]. Dynamic recrystallization usually causes better grain refinement. Using severe plastic deformation techniques (SPD), ultrafine grain size around 1 μm can be achieved [25].

It is well known that producing a fine-grained microstructure in metal improves its mechanical properties [26,27]. Nanostructured materials can be created using techniques based on SPD, such as, e.g., equal channel angular pressing (ECAP), grinding, or modified rolling [28,29,30,31]. The reduction of grain size is accompanied by other microstructural changes, e.g., the introduction of internal stresses, texture development, or increase in density of crystal lattice defects [32]. Surface mechanical attrition treatment (SMAT) is another SPD method that can produce a hard layer of gradient nanocrystalline microstructure in the treated surface [33,34]. The method is based on the same principle as a conventional shot peening. The SMAT process is based on surface plastic deformation induced by repeated impacts on the treated surface with high-velocity spherical shots of a few millimeters in diameter. The shots randomly hit the sample surface, which causes local plastic deformation. Multidirectional impacts induce grain refinement to the nanometer scale at the target surface. Formation of a nanostructured layer as a result of SMAT has been reported for many metals and alloys, e.g., stainless steel, aluminum, copper, and titanium [35,36,37,38,39,40,41].

It is generally believed that the microstructure refinement improves the corrosion resistance of magnesium and its alloys [42]. This applies not only to SMAT but also to other techniques of the surface treatment (e.g., severe shot peening [43] and surface burnishing [44,45,46]. However, the deformation amount is important and reports showing the opposite result of the corrosion properties for the same/or very similar surface treatment technique are available (e.g., Reference [47]).The outcome of the SMAT on the corrosion resistance depends on the nature of the material and the mechanical treatment parameters [38,39]. There are reports of decreasing corrosion rate, e.g., titanium or aluminum show a reduced corrosion rate after SMAT [48,49]. On the other hand, Chen et al. and Li et al. observed the detrimental influence of SMAT on the corrosion resistance of AZ31 magnesium alloy [50,51]. The same behavior was observed in pure magnesium by Fabijanic et al. [39]. Similar discrepancies also apply to the effect of other SPD methods on corrosion properties, e.g., the detrimental impact of the ECAP on the corrosion resistance of pure magnesium shown by Song et al. and an increase in the corrosion resistance of pure magnesium due to high-pressure torsion (HPT) shown by Silva et al. [52,53].

The gradient microstructure created by SMAT has been widely investigated using standard experimental techniques, e.g., optical, scanning, and transmission electron microscopy, X-ray, and electron diffraction [54]. Nevertheless, positron annihilation spectroscopy (PAS) as a method of studies of defects in a crystalline solid matter at an atomic level has rarely been applied for this purpose [55,56]. PAS is a suitable tool for detecting open volume defects created during plastic deformation induced by various mechanical treatments [57,58,59]. There are several advantages to using this method. In general, it is non-destructive and extremely sensitive to the presence of crystal lattice defects in materials studied. It allows tracing microstructural changes in materials after plastic deformation. Before annihilating with electrons, positrons probe a certain depth inside the target material depending on its density. Thus, the information about microstructure is averaged over some depth of the order of tens of micrometers. If the subject of interest is a very thin layer (up to 1 µm) located just below the surface of the material, the Variable Energy Positron (VEP) beam technique can be used [60].

The goal of the present studies is to characterize commercial purity magnesium subjected to SMAT and to relate the obtained results to corrosion properties determined by electrochemical tests. Positron annihilation spectroscopy (PALS and VEP), X-ray diffraction (XRD), electron backscatter diffraction (EBSD), microhardness tests, and the surface profile characterization were applied to characterize the influence of SMAT on the microstructure of the surface layer. 

## 2. Materials and Methods

### 2.1. Sample Preparation

The specimen 100 mm × 100 mm × 7.6 mm in dimensions was cut from a plate of commercial grade magnesium (99.5% purity). Prior to SMAT, the specimen was annealed at 400 °C in vacuum (~10^–3^ Pa) for half an hour and then slowly cooled inside the furnace to room temperature. In this way, a material with only residual crystal lattice defects was obtained. SMAT was performed in the stainless-steel chamber at room temperature in air using 2 mm diameter stainless steel balls and a vibration frequency of 20 kHz. In order to examine changes of the microstructure induced in the initial stage of SMAT, the two opposite sides of the Mg plate were treated separately for 60 s and 120 s, with vibration amplitudes 13 ± 2 µm and 27 ± 2 µm, respectively. Increasing both the SMAT duration and the vibration amplitude causes an increase in the energy imparted into the treated surface. The vibration amplitude controls the normal speed of the impacts. A higher amplitude leads to a higher impact velocity [61]. In this case, doubling the vibration amplitude will lead to doubling the velocity of the shots, i.e., to approximately factor 4 in the kinetic energy increase. After SMAT, the specimen was cut into smaller pieces 10 mm × 10 mm in size. Pieces of the plate, not subjected to the SMAT process, were kept as a reference. Additional samples prepared from annealed magnesium were deformed at room temperature using a uniaxial hydraulic press to obtain 10%, 23%, and 40% thickness reduction.

### 2.2. X-ray Diffraction Measurements

The X-ray diffraction was carried out using a PANalytical Empyrean diffractometer (by Malvern Panalytical Co., Almelo, the Netherlands and Malvern, UK) with Cu Kα radiation and a parallel beam geometry (Göbel mirror in the incident beam optics and parallel plate collimator in the diffracted beam optics) over the 2θ range of 20–120°, step 0.02°, at room temperature. Diffraction patterns were collected using the multi-reflection grazing-incidence X-ray diffraction method (MGIXD) with 3 different incident angles: 5°, 10°, and 15° [62,63].

### 2.3. Optical Microscopy and Electron Backscatter Diffraction

In order to observe the microstructure, the samples were cross-sectioned, mounted in cold mounting resin, ground with a series of SiC papers down to a grid 2400, and then further polished using aluminum oxides with grain size down to 0.05 μm and the 0.04 μm silica oxide, and, finally, argon ion beam cleaned using an IM4000Plus instrument (by Hitachi High-Tech Corporation, Tokyo, Japan). An optical microscopy Axio Imager M1m (by ZEISS, Oberkohen, Germany) and a Nova NanoSEM 450 Scanning Electron Microscopy (SEM) (by FEI Co, Hillsboro, OR, USA) were used. To reveal the possible presence of iron on the surface of the samples, the SEM-EDS (Energy Dispersive Spectroscopy) analysis using S-3500N microscope (by Hitachi High-Tech Corporation, Tokyo, Japan) with the analyzer NORAN 986B-1SPS (by Thermo Fisher Scientific, Waltham, MA, USA) was performed prior and after SMAT.

### 2.4. Micro-Hardness Tests and Surface Characterization

The microhardness measurements were performed using a 2500 Instron ITW device (by Instron, Norwood, MA, USA) with a Knoop indenter with a maximum load of 0.01 kg (HK0.01). The results were converted into Vickers hardness. During measurements, the indenter longer diagonal was parallel to the trace of the SMATed surface. Surfaces of SMATed magnesium samples were characterized using an optical profiler WYKO NT9300 (by Veeco, New York, NY, USA).

### 2.5. Positron Annihilation Spectroscopy

PALS measurements were conducted using a fast-fast spectrometer with BaF_2_ scintillators (photomultipliers XP2020Q manufactured by Photonis, Brive, France and other electronic components made by ORTEC, Oak Ridge, TN, USA). The time resolution of the spectrometer was about 280 ps (full width at half maximum). The source of positrons containing ^22^Na isotope enveloped into a 7 μm thick Kapton foil was sandwiched between two identical magnesium samples placed in front of the scintillator detectors of the spectrometer. All the obtained spectra containing more than 10^6^ counts were analyzed using the LT code, taking into account the source contribution and background subtraction [64].

Positrons emitted from the radioactive ^22^Na isotope have a continuous energy spectrum; thus, they probe a certain depth inside the target material. The linear absorption coefficient µ for positrons from the ^22^Na source implanted in magnesium is equal to ca. 1/130 µm^−1^. Therefore, the contribution to the measured value of the positron lifetime comes from a layer with a thickness comparable to 1/μ. Hence, it is possible to obtain a depth profile of the positron lifetime by sequential removal of layers of the sample. In our studies, thin layers of samples were removed by sequential etching in Nital and PALS measurements were carried out. After each step, the thickness of the removed layer was measured using a digital micrometer caliper with ±1 µm accuracy. Due to the penetration range of positrons from the ^22^Na source, much larger than the range of additional defects that could be introduced by etching, we assume that chemical removal of the sample layers does not affect the measured positron lifetime values [65,66].

VEP beam was used to study microstructure changes of the near surface region of the magnesium samples subjected to SMAT. The method allows implanting positrons inside the target material to the depth in the range of single nanometers to a few µm. The positrons were formed into a mono-energetic beam with an intensity of ca 10^5^ e^+^/s and a diameter of 5 mm. The energy of implanted positrons ranged from 0.2 to 26 keV. The behavior of implanted positrons in solids is described by diffusion theory in which the positron diffusion length is a crucial parameter. It depends on the properties of the material and defects present inside it [67].

The Doppler broadening spectrometer (by ORTEC, Oak Ridge, TN, USA) with HPGe (High Purity Germanium) detector of resolution 1.2 keV at 511 keV was used to register positron annihilation quanta. The registered annihilation photons convey the information on the local electron momentum distribution, which changes in a specific way. These changes are reflected in the shape of the annihilation peak, which can be characterized by the so-called *S*- and *W*-parameters [67]. The *S*-parameter demonstrates the contribution of positrons annihilating with low momentum electrons present mostly in open volume defects. The *W*-parameter represents the contribution from annihilation with core electrons, which have higher momenta. In general, the values of the *S*- and *W*-parameters measured for a given positron incident energy are weighted averages of their values for positron annihilation from different states: free positrons, positrons trapped in defects inside the material, and positrons annihilating from surface-related states. Detailed information about the used VEP beam can be found elsewhere [60].

### 2.6. Corrosion Tests

Corrosion tests were performed in an all glass-and-PTFE (Polytetrafluoroethylene) cell with a platinum foil as a counter electrode and the saturated calomel electrode (SCE) as a reference electrode. All potentials are reported versus that electrode. Tests were performed in a 0.15 mol dm^–3^ NaCl solution, saturated with Mg(OH)_2_. The composition of that solution is close to the solutions in which magnesium objects corrode in practical applications: in the human body (implants) and in the land transportation (due to the spilling of sodium chloride on the road surfaces in winter). Magnesium hydroxide is formed during the magnesium corrosion process due to the reaction: Mg + 2H_2_O = Mg(OH)_2_ + H_2_. The pre-saturation of the test solution with Mg(OH)_2_ stabilizes not only the composition of the solution but also pH, which is very important because the corrosion rate of magnesium strongly depends on pH. Note that the solubility of magnesium hydroxide in water is very low (*K_sp_* = 5.61 × 10^−12^).

The working electrode with an exposed area of 0.25 cm^2^ was placed at the bottom of the cell, in a horizontal position, with the active surface up to facilitate liberation of the hydrogen formed in the corrosion process. Before the measurement, the solution was bubbled with argon to remove dissolved oxygen. The measurements were carried out at room temperature 21 ± 1 °C. The measurements started with 2 h registration of the open circuit potential (OCP). Then the linear sweep voltammetry (LSV) was performed to trace the polarization curve, starting from the potential of −0.2 V versus OCP in the anodic direction with the potential sweep rate of 1 mVs^−^^1^. Polarization was continued up to the attainment of the current density of ~10 mA cm^−^^2^. In some cases, LSV was preceded by the registration of the electrochemical impedance spectroscopy (EIS) spectrum in the frequency range of 10 kHz—10 mHz and the amplitude of the sinusoidal voltage signal of 10 mV. Gamry G300 Potentiostat/Galvanostat/ZRA (by Gamry Instruments, Warminster, PN, USA) or Autolab PGSTAT 302N (by Metrohm Autolab B.V., Utrecht, The Netherlands) were used in the experiments.

## 3. Results and Discussion

### 3.1. Surface Characterization

**Figure 1:**
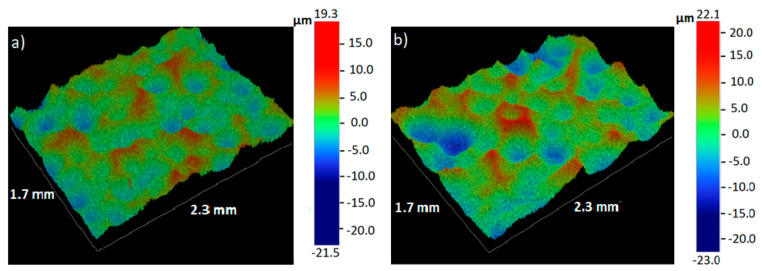
Optical profiler images for magnesium samples surface mechanical attrition treatment (SMAT)ed for 60 s (**a**) and 120 s (**b**).

Figure 1 presents the surface morphology of magnesium samples after 60 s and 120 s of SMAT. On both surfaces, there are visible craters formed due to the impacts of steel shots during the treatment. The roughness parameter *R_a_* increased from the initial value of about 1 μm to 3.37 μm and 4.63 μm for 60 s and 120 s of SMAT duration, respectively. It is known that the extent of the roughness increase in SMAT depends on the ball type and size, the treatment time, and the vibration amplitude [68,69]. The higher roughness caused by the higher vibration amplitude was reported, e.g., for stainless steel [70]. The surface roughness values obtained in this study are similar to those obtained by Laleh and Kargar for AZ91D alloy SMATed for 30 min with stainless steel balls with a similar diameter [38]. However, they are higher than those reported by Li et al. for pure magnesium and Mg–1Ca alloy SMATed with zirconia balls (2 mm in diameter) over a range of times from 40 to 90 min [21]. It is worth mentioning that the dependence of the surface roughness on the SMAT duration does not necessarily have to be monotonic. Kargar et al. reported that the surface roughness of copper initially increased sharply and decreased slightly with the increasing treatment time from 10 to 300 min [71].

### 3.2. Microstructure

**Figure 2:**
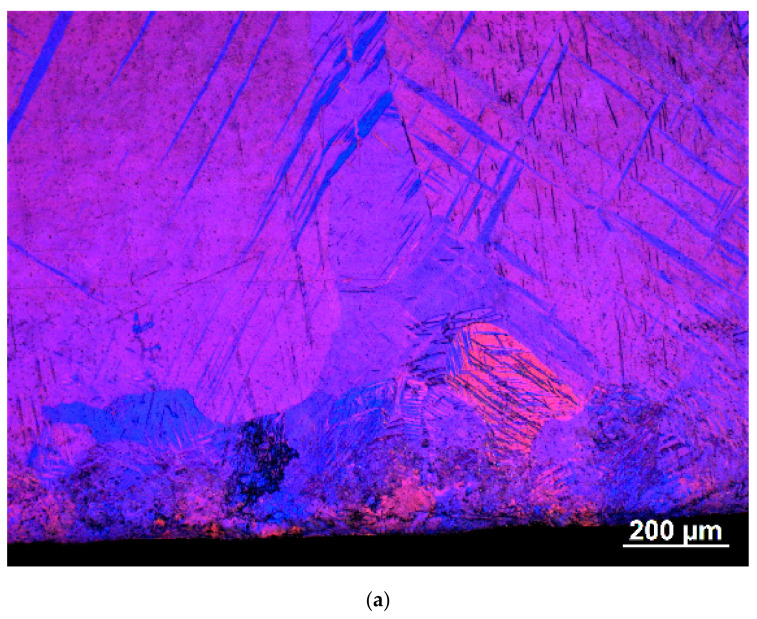

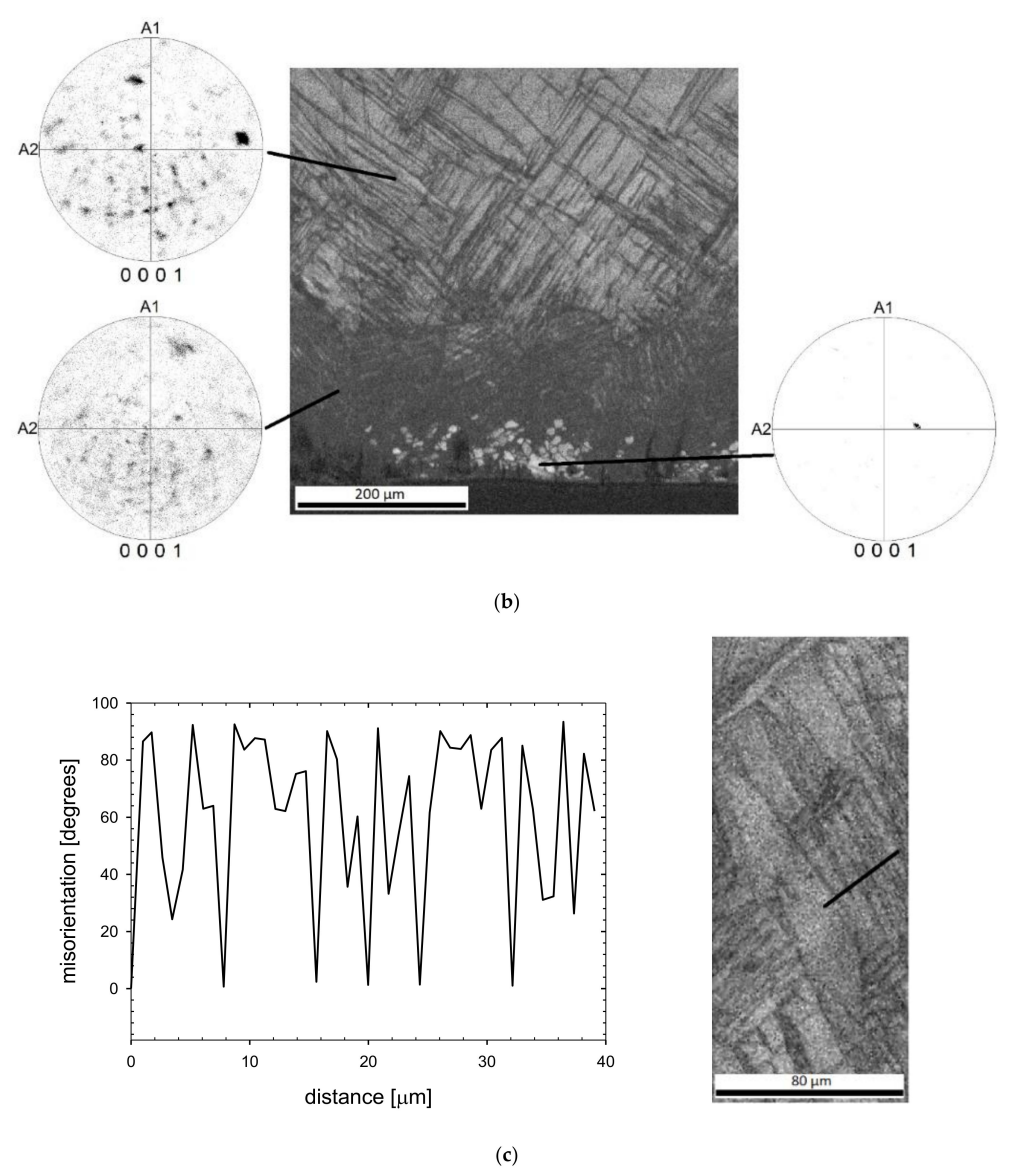
A typical microstructure on the cross-section of the sample SMATed for 120 s (SMATed surface can be seen in the bottom): (**a**) optical microscopy, polarized light, (**b**) the Image Quality Microstructure reconstructed from electron backscatter diffraction (EBSD) measurements and corresponding local crystallographic orientations shown in 0001 pole figures, and (**c**) magnification of a selected subsurface area with corresponding misorientations along the marked black line.

The microstructure was examined using optical microscopy with polarized light and the EBSD technique. These studies were performed on the sample cross-sections perpendicular to the SMATed surface. Typical images, shown in Figure 2, were selected from results obtained for the sample treated for 120 s. A significant decrease in the grain size and the presence of characteristic twins belonging to different twinning systems in which density gradually decreases with a distance from the treated surface can be seen. The SMAT affected microstructure extends to a depth of about 800–900 μm. This result corresponds well to the thickness of the hardened layer (see Section 3.3). Directly at the surface up to the depth of 200 µm, the density of twins is very high. In this subsurface layer up to the depth of about 80 µm, some very small twin free grains are present. Based on EBSD results, their size can be estimated as less than 10 μm. The presence of finer grains cannot be excluded, but their unambiguous identification requires a more resolved research technique (i.e., transmission electron microscope). The same type of microstructure was formed in the sample treated for 60 s, but, in this case, the fine twin free grains were not observed. Another difference concerns the estimated density of the twins and the SMAT affected layer’s depth, which was smaller for shorter SMAT duration with the lower vibration amplitude due to the lower energy imparted to the surface. The same type of microstructure was reported in the literature for some SMATed metals with the hexagonal close-packed structure (hcp) [72,73,74,75].

At a high strain rate at room temperature, mechanical twinning is the main deformation mode of hcp metals, such as magnesium and titanium, especially when the strain is not too high [75]. Twinning can be responsible for the grain refinement and results in strain hardening. Twins produce characteristic misorientations of the crystal lattice, and such misorientations were used in the present research to identify twins (see Figure 2) [76].

One can expect that the SMAT induced heat effect can result locally in recrystallization of highly deformed material, which causes formation of small recrystallized grains, as observed in the present research. Recrystallization nuclei and very fine recrystallized grains usually do not contain recrystallization twins, or the density of such twins is relatively low. Therefore, our observation of the small twin free recrystallized grains is understandable. Such grain formation can be related to a very low recrystallization temperature of pure magnesium. As it was found by Ichikawa, recrystallization of magnesium can start already at 75–175 °C [77]. Thermomechanical simulations performed by Rouquette et al. indicate that the temperature can reach locally 200 °C during shot impact [78]. The occurrence of these grains limited to a very thin region close to the SMATed surface and only for the 120 s treated material indicates that the thermal effect of SMAT is weaker than the strengthening one and that 60 s treatment with the lower vibration amplitude is not sufficient to induce both of them strongly. This explanation is confirmed by the results of the microhardness measurements (see Section 3.3).

The XRD method was used to determine the effect of SMAT on the internal microstructure of the topmost layer of the samples. The broadening of the diffraction peaks measured in the symmetrical diffraction mode was analyzed compared to the reference sample. To calculate the average crystallite size, the well-known Williamson-Hall (W-H) method was applied:

(1) $$\beta \cos\theta =\frac{k\lambda }{l}+\eta \sin\theta ,$$

where *β* is full-width at half maximum of the peak corrected for the instrumental broadening, *θ* is the diffraction angle, *k* is constant (0.9), *λ* is the X-ray wavelength, *l* is the size of the coherent diffraction domains, which can be related to the notion of crystallite (i.e., crystal volumes relatively free from defects), and *η* is linked to the root-mean-square lattice strain [79]. Crystallites are usually smaller than the dislocation cells or the subgrains observed by transmission electron microscopy. The crystallite is defined as a very low dislocation density volume bounded by an area with significantly increased dislocation density and much smaller than the grain, especially for the plastically deformed materials [80,81]. In the calculations, all registered peaks from the XRD patterns were used. The values of the crystallite sizes are gathered in Table 1. The W-H analysis results show that SMAT significantly reduces the size of the crystallites in comparison to the reference sample. The extension of the SMAT duration from 60 s to 120 s and doubling the vibration amplitude do not considerably change the crystallite size. Crystallites of comparable size were found in the case of 40% thickness reduction by uniaxial pressing, while, for 10% thickness reduction, slightly larger crystallites were obtained. Li et al. reported similar crystallite size for pure magnesium subjected to SMAT [21]. The lattice strain, which can be related to crystal lattice defect density, is comparable to the error of the W-H method. It was equal to 0.01(1)% for the reference sample, the sample SMATed for 60 s, and the compressed sample with 10% thickness reduction. For the sample treated for 120 s and the pressed one with 40% thickness reduction, the lattice strain was slightly higher (i.e., at least 0.02(1)%). 

### 3.3. Microhardness

**Figure 3:**
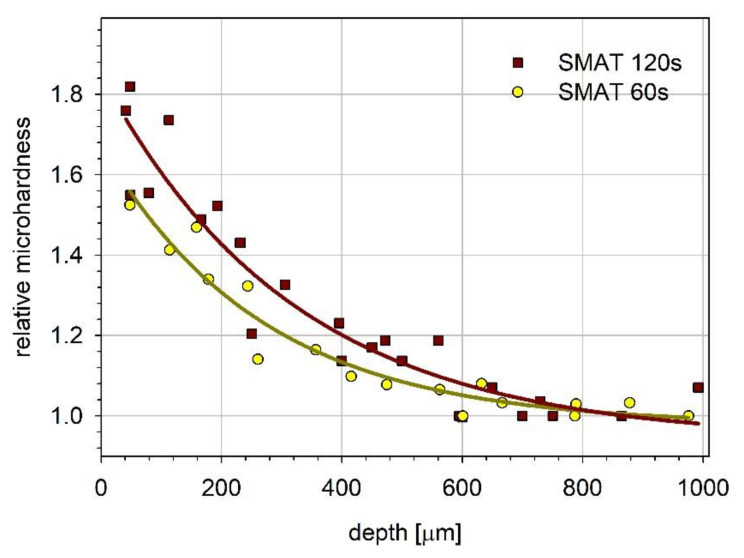
The relative microhardness of magnesium samples SMATed for 120 and 60 s versus depth. The solid lines represent the best fit of the exponential decay function to the experimental points: $HK_{60}\left(z\right)=0.98+0.7\exp\left(-z/268\right)$ and $HK_{120}\left(z\right)=0.94+0.9\exp\left(-z/322\right)$ for 60 s and 120 s-SMATed samples, respectively; z is the depth beneath the surface.

Figure 3 shows the microhardness changes with the distance from the SMATed surface. The microhardness measured at a depth of about 40 μm for the samples treated for 120 s and 60 s is, respectively, 1.8 and 1.55 times higher than the value for the reference material. The microhardness gradually decreases with the distance from the surface, reaching the reference value at a depth of about 800–900 μm. The obtained dependencies of the microhardness on the depth can be described by the exponential decay function. The decay length, which determines the depth at which the microhardness drops to 1/e of its initial value, is equal to 268 μm and 322 μm for the treatment time of 60 s and 120 s, respectively. The difference between these values is comparable with their uncertainties, and there is not distinguishable variation in the total thickness of the deformed layer for the two treatment regimes.

Literature data on the hardness profile for SMATed pure magnesium is relatively rare. Fortunately, data for magnesium alloys are much more frequent. All of them usually show that SMAT treatment generates some hardness gradient at the treated surface similar to that observed in the present study. The same applies to other metals at least with hcp and fcc (face centered cubic) structure regardless of the stacking fault energy, e.g. reference [82,83]. A slightly hardened surface layer (an increase in hardness reaching 20%) with a thickness of 1.75 mm was observed in pure magnesium. Mg-1Ca, treated in the same way, developed a hardened layer 1 mm thick with the increase the hardness reaching 80% [21]. One can see that the hardness profile determined in the present research is very close to that observed by Li et al. for the Mg-1Ca alloy [21]. A similar increase in microhardness was observed for Mg-6Gd-3Y-0.5Zr alloy for the SMAT duration of 3 min, and the hardened layer thickness was 1.2 mm [84].

The results of the present work show that the SMAT duration and the vibration amplitude affect the microhardness profile causing its increase by about 55% to 80% of the initial value and the differences inside the material. This confirms that increasing the energy imparted to the surface by increasing vibration amplitude and/or treatment duration leads to an increase in the material hardness deeper in the material [69]. However, the literature data indicate that, for much longer SMAT, the influence of its duration is weak as reported for Mg, Mg-1Ca alloy, and the AZ31 alloy [21,85]. For shorter SMAT times of the AZ31B alloy (5–15 min), the effect was more pronounced [72]. A two or three–fold extension of the SMAT time resulted in an approximate increase in hardness from 70% to 120%, comparable to our results.

### 3.4. Residual Stress

No significant effect of SMAT (regardless of the process duration and the vibration amplitude) on residual stress was found. Using the MGIXD method, it was possible to determine residual stresses for 3 different penetration depths: 12 µm (for α = 5°), 21 µm (for α = 10°), and 29 µm (for α = 15°). The value of residual stresses is smaller than the uncertainty so that it can be considered equal to 0, and it remains constant with the depth in the sample (up to 30 µm). This result is consistent with the present results of the microstructure studies that show that recrystallized grains extend far deeper below the SMATed surface than the depth of the stress measurement (see Figure 2). It is well known that recrystallization is preceded by stress relief during recovery [86,87]. Fabijanic et al. also reported relatively low compressive residual stresses of 7.1 ± 2.8 MPa, 6.5 ± 2.6 MPa, and 12.4 ± 4.6 MPa, for magnesium treated using steel, alumina and zirconia balls, respectively [39]. However, the differences in the treatment parameters in comparison to the present study, such as the ball diameters (i.e., 11 mm, 8 mm, and 10 mm, for alumina, steel, and zirconia balls, respectively), the vibration frequency of the system (50 Hz), and treatment time (60 min) should be mentioned. Low residual stress in SMATed magnesium corresponds to very low lattice strain value, i.e., below 0.06%, as determined by Li et al. [21]. Those authors also noted that up to 60 min of SMAT, the lattice strain increased, but the next 30 min of SMAT caused its significant decrease. It is worth mentioning that Li et al. used 2 mm zirconia balls [21]. Taking into account the influence of the ball material on residual stresses reported by Fabijanic et al., zirconia balls would cause significantly higher residual stresses than the steel balls used in the present work.

Higher residual stress can be developed in the magnesium alloys due to SMAT and similar surface treatments. However, even in severely shot-peened AZ31 magnesium alloy, the maximum value of residual stress is only about −50 ± 10 MPa and occurred under the treated surface solely at a depth of 100 µm. Below and above that depth, the stress dropped to near zero [88]. Liu et al. also reported that in the 3 min SMATed GW63K magnesium alloy, the lattice strain was about 0.1% [84]. Relatively small residual stress values in SMAT treated magnesium and its alloys can certainly be associated with their easy recrystallization due to the low melting point. In other hcp metals with a higher melting point, such as titanium and zirconium, these stresses are also compressive but much higher (e.g., several hundred MPa) [89,90,91,92].

### 3.5. Positron Lifetime Measurements

**Figure 4:**
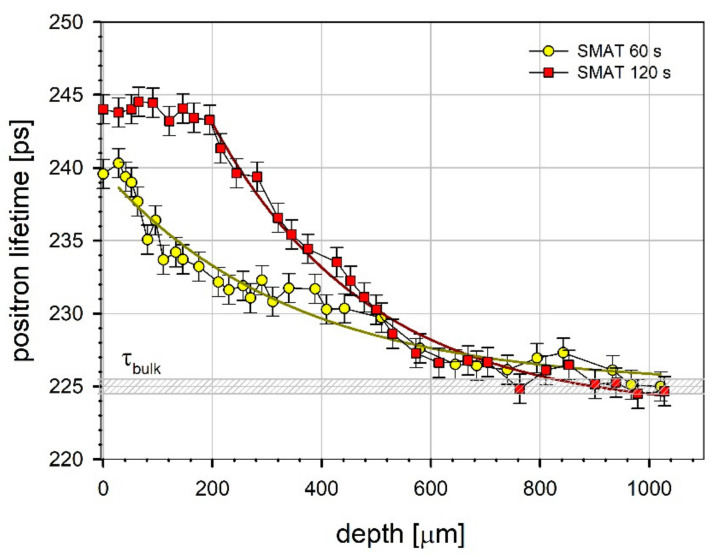
The mean positron lifetime profiles. The solid lines represent the best fit of the exponential decay function to the experimental points: ${\bar{\tau }}_{bulk}\left(\mathrm{in}\mathrm{ps}\right)=225+13.6\exp\left[-\left(z-28\right)/342.8\right]$ for the 60 s-SMATed sample and ${\bar{\tau }}_{bulk}=223+20.2\exp\left[-\left(z-195\right)/285.7\right]$ for the 120 s-SMATed sample, where z is the depth from the treated surface in μm.

Only one positron lifetime was resolved in the measured positron lifetime spectra. This can be explained by the presence of a variety of crystal lattice defects characterized by not much different lifetime values and the finite time resolution of the spectrometer. This lifetime can be treated as the mean positron lifetime denoted as $\bar{\tau }$. Because the implantation range (see Section 2.5) is smaller than the layer affected during SMAT, it is possible to observe the changes of the registered positron lifetime as a function of the depth from the surface of the sample. Figure 4 presents the mean positron lifetime dependencies on the distance (or depth) from the SMATed surface of the magnesium samples. The hatched rectangle represents the value of the positron lifetime obtained for the reference sample, $\tau_{bulk}$ = 225 ± 1 ps, which is in agreement with that obtained by Hautojärvi et al. [93]. However, a slightly shorter lifetime (222 ps) was also reported [94]. The mean positron lifetime values obtained on the surface are equal to 240 ± 1 ps and 244 ± 1 ps for 60 s and 120 s treatment duration, respectively (the latter with the doubled vibration amplitude). For comparison, the mean positron lifetime values for the samples deformed by compression are equal to 234 ± 1 ps and 241 ± 1 ps for 10% and 40% of thickness reduction, respectively.

For the sample SMATed for 120 s, $\bar{\tau }$ does not change noticeably up to the depth of about 195 µm. It decreases significantly for a depth between 195 µm and 600 µm. Then, $\bar{\tau }$ decreases slowly to reach $\tau_{bulk}$ at about 900 µm. For the sample SMATed for 60 s, the decrease in $\bar{\tau }$ starts close to the surface and reaches $\tau_{bulk}$ for the depth slightly larger than 900 µm. The solid lines represent the best fit of the exponential decay function to the experimental points. The depth dependencies obtained for the mean positron lifetime and microhardness (Figure 3) exhibit similarities. Both quantities take the highest values at the surface of the sample SMATed for 120 s. The total range of the observed changes is also similar. However, the mean positron lifetime is averaged over some volume of the sample, which reduces the scattering of the measurement points.

The mean positron lifetime values close to the surface for both SMATed samples are much higher than the bulk value, indicating the presence of a layer with a high density of crystal lattice defects introduced by SMAT. Generally, dislocations introduced by plastic deformation in metals are shallow traps for positrons, but they are usually accompanied by a high number of jogs and associated vacancies. Those defects can act as deeper positron traps. For a high degree of deformation in magnesium, multiple slip systems are activated, and the interaction of moving dislocations introduces jogs on their lines. Trapping of positrons by twin boundaries should also be taken into account. It has been reported by Serra and de Diego for deformed titanium [95]. These authors concluded that dislocations associated with twin boundaries could act as positron traps with lifetimes lower than those of pure lattice dislocations. This is because vacancies and dislocations tend to minimize their open volume when localized in twin boundaries. Thus, twins, dislocations, dislocations jogs, and adjacent vacancies could be responsible for trapping positrons in deformed magnesium samples. All this causes that a reliable analysis of the positron lifetime spectra is performed in terms of the mean positron lifetime, even though, in some cases, the positron lifetime components coming from the annihilation of positrons trapped by dislocations and jogs could be resolved as was reported in the literature. For magnesium deformed by compression, del Río et al. ascribed the lifetime component of 253 ps to the annihilation of positrons trapped in jogs and 244 ps to positrons trapped in dislocations without jogs [94]. However, the reported mean positron lifetime for samples deformed by compression lower than 253 ps was explained by a contribution of positrons annihilating in undisturbed crystal lattice regions. The values of the positron lifetime in monovacancies reported by Häutojarvi et al. and Folegati et al. are 253 ps and 245 ps, respectively [93,96].

The dependencies obtained for the SMATed samples reflect the defect depth distribution below the treated surface. The plateau of the high $\bar{\tau }$ values extending to about 195 μm coincides with the highly deformed layer (with a high density of twins, see Figure 2). It is not excluded that the visible grains in this layer also contain defects localizing positrons. The decrease of the mean positron lifetime coincides with decreasing density of twins in deeper layers. For the sample SMATed for 60 s, the value of $\bar{\tau }$ at the surface is lower than for the sample SMATed for 120 s, which indicates a lower concentration of defects. In this case, the defect concentration starts to decrease much closer to the surface.

The PALS results for SMATed magnesium can be compared with those for ultra fine grained bulk magnesium obtained by HPT reported by Čížek et al. [97]. In those studies, two lifetime components in the positron lifetime spectrum were resolved. The longer lifetime, equal to 257 ± 3 ps, was ascribed to the presence of vacancies associated with dislocations. The shorter lifetime, equal to 188 ± 5 ps, significantly lower than the bulk value, indicated the presence of regions recrystallized as a result of dynamic recrystallization (DRX). Its intensity, 39%, was quite high. It should be noted that the mean positron lifetime calculated as a weighted average of these two components is equal to 230 ps, which is definitely lower than the mean positron lifetime at the SMATed surface even for only 60 s SMAT duration, indicating a lower defect density. According to Sun et al., in comparison to other severe plastic deformation processes, such as HPT or ECAP, the strain rate in SMAT is much higher, which leads to obtaining smaller refined grains [98,99]. As it was shown by Okhubo et al., the strain rate can also influence the generated crystal lattice defects [100].

The mean positron lifetime values similar to those for the SMATed magnesium were obtained for magnesium subjected to dry sliding against stainless steel [66,101]. This is understandable, taking into account that sliding friction involves large amounts of near-surface plastic deformation. Nonetheless, a plateau with high values of the mean positron lifetime beneath the worn surface was not observed for the relatively short sliding distances in the reported studies. The obtained dependencies of $\bar{\tau }$ on the depth were described by the exponential decay function, and its decrease started at the surface as for the sample SMATed for 60 s. It is worth noticing that similar plateaus beneath a few micrometer thick mechanically mixed layer were observed for other metals and alloys for longer sliding distances [102]. Their presence was explained by the accumulation of deformation for repeating passages of a sample over a hard counterface. However, for both processes: SMAT and sliding friction, a gradient of the defect concentration occurs in the subsurface region.

### 3.6. Variable Energy Positron Beam Measurements

**Figure 5:**
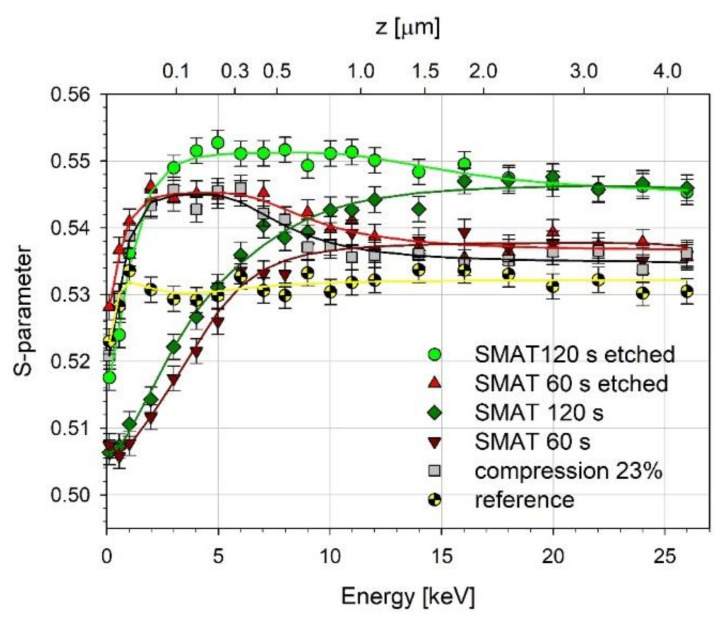
The *S*-parameter as a function of incident positron energy (mean implantation depth).

Figure 5 shows the *S*-parameter as a function of the positron incident energy measured for the samples SMATed for 120 s and 60 s and the same samples after etching in Nital. For the sake of comparison, the same dependency for the reference sample and the sample compressed to 23% thickness reduction then etched in Nital are also shown. The top horizontal axis denotes the mean implantation depth of the annihilating positrons calculated using the formula $\bar{z}$ (nm) = 1.4*E*^1.757^, where *E* is the positron incident energy (in keV) [103]. According to the literature, the lower values of the *S*-parameter for low positron incident energy observed for all samples indicate the presence of an oxide layer on the surface of the metal [104,105,106,107]. For the SMATed samples without etching, the *S*-parameter increases with the increase of the incident positron energy and saturates. The *S*-parameter saturated value and the positron energy at which the *S*-parameter saturates differ for these two samples. For the 60 s SMATed sample, the *S*-parameter saturates at about 7 keV, while, for the 120 s SMATed sample, it saturates at higher energy, even close to 12 keV. We postulate that, up to these energies, positrons penetrate mainly the oxide layer. For higher positron energy, the metal matrix is penetrated. The higher saturated value of the *S*-parameter for the 120 s SMATed sample than that for the 60 s SMATed sample is in agreement with the higher value of the mean positron lifetime measured using the conventional PALS method and indicates a higher concentration of positron trapping defects induced by SMAT in the metal matrix.

To confirm this, we etched the samples in Nital to remove the oxide layer, and the measurement was repeated. The additional measurement was performed for the sample compressed to the thickness reduction of 23%. Note, however, that due to the high reactivity of magnesium towards oxygen, a thin oxide layer will be spontaneously formed at the surface after removal of the sample from the solution. All etched samples exhibit the *S*-parameter dependencies of a similar shape, which significantly differ from those obtained for the samples before etching. The *S*-parameter values at the surface are higher than before etching. The *S*-parameter increases for the positron energy up to 5 keV for the 120 s SMATed sample and 2 keV for the 60 s SMATed and compressed samples. There is a visible broad maximum, after which the *S*-parameter decreases to the saturated values obtained for the samples before etching. Therefore, one can conclude that the saturated values of the *S*-parameter correspond to the annihilation of positrons in the metal matrix of the samples. The *S*-parameter values higher than those for the metal matrix of the samples may indicate the presence of open volume defects induced by etching, as shown, for example, for aluminum [104,105]. The broader maximum and the higher values of the *S*-parameter for the sample SMATed 120 s compared with the sample SMATed 60 s suggests a higher concentration of defects and, therefore, a higher reactivity of the former sample.

For a given positron energy *E* (i.e., penetration depth), the measured *S*-parameter can be expressed as

*S* = *F_s_S_s_* + *F_ox_S_ox_* + *F_id_S_id_* + *F_m_S_m_*, (2)

where *F_s_*, *F_ox_*, *F_id_*, and *F_m_* are the fractions of positrons annihilated at the surface, in an oxide film, at defects (including the oxide film/matrix interface and pores, etc.), and the metal matrix, and *S_s_*, *S_ox_*, *S_id_*, and *S_m_* are the corresponding values of the *S*-parameter in these regions. The dependencies *S*(*E*) can be fitted using the standard VEPFIT code, which solves the diffusion equation taking into account the positron implantation profile [108]. The results are solid lines in Figure 5. The fits were obtained assuming that positron diffusion length in the metal matrix of the samples is much shorter than diffusion length in annealed defect-free magnesium, which is close to 200 nm [109]. For example, Hruška et al. reported the positron diffusion length in Mg films prepared by RF (radio frequency) magnetron sputtering at room temperature 35–45 nm [110]. In the case of those Mg films, the shortening of the positron diffusion length is caused by defects, such as misfit dislocations necessary for the accommodation of the lattice mismatch between the film and the substrate and open volume point defects (vacancies and vacancy clusters) located at grain boundaries. We assumed that the positron diffusion length in the metal matrix of the SMATed samples has similar or lower values due to the severe plastic deformation. This assumption allowed us to obtain the positron diffusion length in the oxide layer for the SMATed samples. For both SMATed samples before etching, the positron diffusion length has a higher value, about 74 nm. This value is comparable to those reported by Yang et al. for the layer of the corrosion products for AM60B magnesium alloy immersed in NaCl solution for longer immersion times and higher thickness of the layer [106]. The thickness of the oxide layer for the SMATed and reference samples (Table 2) correlates with their corrosion resistance estimated by LSV. The increase of the oxide layer thickness with the SMAT time indicates that SMAT increases the susceptibility to oxidation (gas corrosion).

For the etched samples, we obtained the thickness of the layer containing defects induced by etching, assuming that the thickness of the oxide layer on the surface is much lower than before etching. The positron diffusion length in the layer for the etched samples is very short, about 10 nm, which confirms a high concentration of defects. The thickness of the defect layer increases with the increase of the SMAT duration and the vibration amplitude.

**Figure 6:**
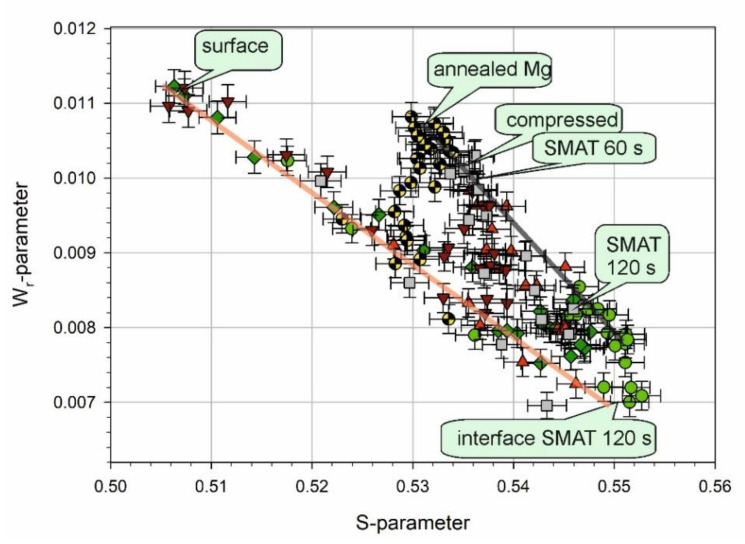
*W_r_*-parameter as a function of *S*-parameter. The symbols are the same as in Figure 5.

Information about the type of defects in all samples can be revealed by the *W_r_*- versus *S*-parameter plot, as shown in Figure 6 [67,111]. Generally, a straight-line segment in such plots indicates that only two positron states contribute to annihilation. The end points of this segment correspond to phases or defects having particular *S*- and *W_r_*-parameters. It seems that the points in Figure 5 for which an increase of the *S*-parameter starts at the surface lie along a straight line in Figure 6. For higher values of the positron incident energy, the points deviate from this straight line. Yang et al. observed similar behavior for AM60B magnesium alloy after immersion in NaCl solution for the initial sample and short immersion times [106]. It was explained as caused initially by the presence of an oxide layer and then a corrosion product layer, whose thickness increases with the increase of the immersion time, and an interface between this layer and the metal matrix. In our case, the states from which positrons annihilate in the metal matrix of the samples SMATed for 60 s and 120 s, and the compressed sample may differ because of different kind and amplitude of deformation and possibly different kind and density of defects. However, it can be seen that the points for which the *S*-parameter values saturate in Figure 5 are gathered in three locations in Figure 6: The first is described as bulk for the 120 s SMATed sample, second as bulk for the 60 s SMATed sample, and third as bulk for the compressed sample. The difference between the two latter is small. Furthermore, these three locations and the points for annealed Mg also lie along another straight line, which suggests that defects in the metal matrices of the samples are similar, and only their concentration changes.

### 3.7. Corrosion Resistance

Magnesium is a “difficult case” in corrosion research, mainly due to its high susceptibility to corrosion, which causes the properties of the measured sample to change during the measurement. The corrosion rate of magnesium is not only high, but it also changes significantly with time. Such behavior was described by many researchers (see, for example, the papers of Qiao et al. and Shi et al.) [112,113].

**Figure 7:**
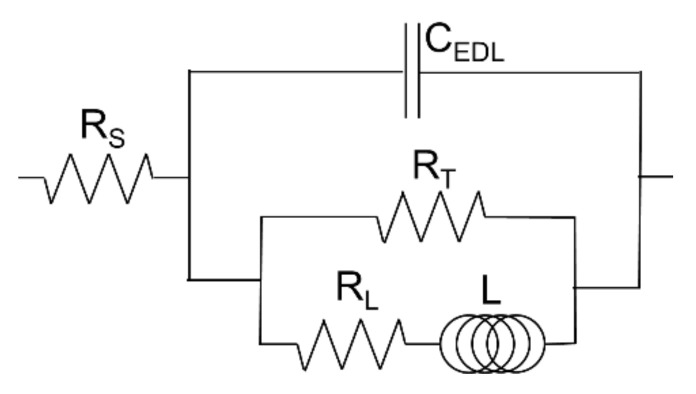
Equivalent electrical circuit applied in the interpretation of the data from electrochemical impedance spectroscopy measurements. *R*_S_ = resistance of the solution, C_EDL_ = capacitance of the electrical double layer, *R*_T_ = charge-transfer resistance, L = inductance of the electrode process, *R*_L_ = resistance connected with the inductance of the electrode process.

**Figure 8:**
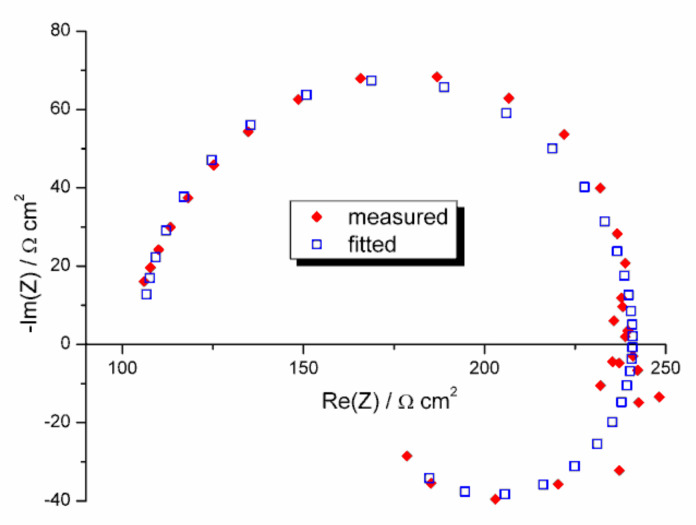
An example of the fit of the equivalent electrical circuit (EEC) showed in Figure 7 to the impedance data. Mg not subjected to any treatment (reference sample) in 0.15 mol dm^–3^ NaCl solution saturated with Mg(OH)_2_.

Three parameters characterizing the corrosion resistance of the investigated samples were calculated from the LSV measurements: corrosion potential, *E*_corr_ (the potential at which the plot *j* = *f*(*E*) crosses the abscissa), polarization resistance (derivative *dE*/*dj* at *E*_corr_), and the corrosion current density, *j*_corr_. Corrosion current density was calculated by the extrapolation of the linear segment on the cathodic branch of the *j* = *f*(*E*) curve traced in the semi-logarithmic coordinates to *E*_corr_ (after correction on the potential drop on the resistance of the electrolyte, which was evaluated in EIS measurements). Note that, according to the Stern-Geary formula, the product of *j*_corr_ and *R*_p_ should be constant for a given material and electrode reaction mechanism, which was roughly fulfilled in our measurements (Table 3). EIS measurements were performed at OCP, and the equivalent electrical circuit showed in Figure 7 was fitted to the impedance data. Figure 8 shows the example of a fit. More information on the data treatment procedure may be found in previous publications of one of the authors [114,115].

For the equivalent electrical circuit (EEC) showed in Figure 7, the polarization resistance, i.e., the parameter characterizing the susceptibility of a material to corrosion, may be calculated from the formula:

(3) $$\frac{1}{R_{p}}=\frac{1}{R_{T}}+\frac{1}{R_{L}}$$

**Figure 9:**
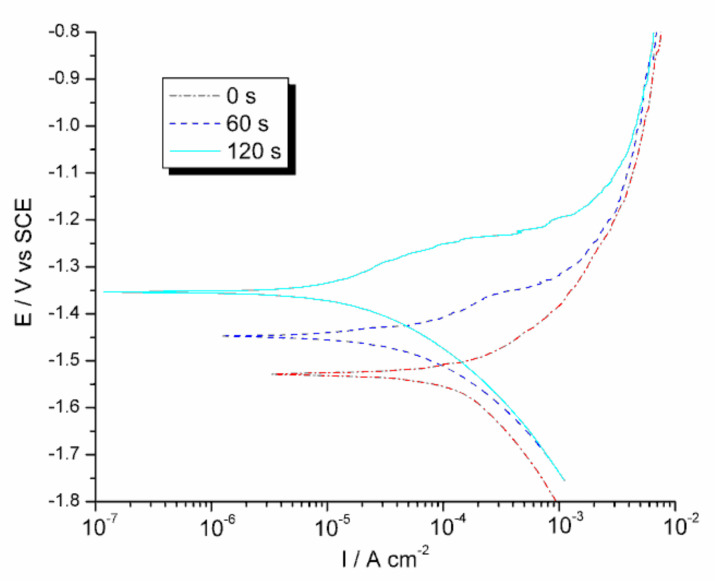
Linear sweep voltammetry on the Mg sample not subjected to any treatment (reference sample), the samples SMATed for 60 s and 120 s in 0.15 mol dm^−3^ NaCl solution saturated with Mg(OH)_2_ at the potential sweep rate of 1 mV s^−1^.

Figure 9 and Table 3 summarize the results of the most representative series of measurements. Three such series of measurements were performed, with some variations in the results (a feature commonly observed in corrosion measurements with nominally identical materials, see, for example, the paper of op’t Hoog [116]); however, the basic characteristics of the material behavior, namely: $E_{\mathrm{corr}}^{0}<E_{\mathrm{corr}}^{60}<E_{\mathrm{corr}}^{120}$, $j_{\mathrm{corr}}^{0}>j_{\mathrm{corr}}^{60}>j_{\mathrm{corr}}^{120}$, and $R_{p}{}_{\mathrm{corr}}^{0}<R_{p}{}_{\mathrm{corr}}^{60}<R_{p}{}_{\mathrm{corr}}^{120}$ were always preserved. The SMAT process strongly influenced the behavior of magnesium in polarization experiments. Although the cathodic branch of the polarization curve practically did not change after the SMAT process (the differences visible in Figure 9 were within the limits of repeatability), the differences in the anodic branch were very significant. Not only *E*_corr_ was shifted in the anodic direction and the magnitude of the corrosion current decreased for the longer SMAT duration with the higher vibration amplitude, but also the shape of the curve changed. A bulge appeared at approximately −1.4 V on the curve traced for the sample SMATed 60 s—even bigger and occurring at higher potential bulge appeared on the curve for the sample SMATed for 120 s. The anodic branches of all three curves show kinks reminiscent of breakdown potential, more numerous the longer the SMAT process was. Notice further that after the attainment of the potential of ~1.0 V the curves coincide with each other, which means that the anodic process is no longer controlled by the surface properties of the sample. At that point, probably transport becomes the controlling factor. All these observations may be explained assuming that SMAT does not decrease but rather increases the susceptibility of magnesium to anodic oxidation, leading to the enhanced formation of hydroxide coverage at the surface and, as a consequence, to the decrease in corrosion current. That point of view is supported by the results of the EIS measurements. For all three samples, OCP was lower than *E*_corr_ measured by LSV, and *R*_p_ measured at OCP by EIS was much lower than the respective *R*_p_ measured at *E*_corr_ in LSV experiments (see Table 3). Moreover, *R*_p_ measured at OCP changed with the energy imparted to the surface (denoted by the SMAT duration) in the opposite direction ($R_{p}{}_{\mathrm{EIS}}^{0}>R_{p}{}_{\mathrm{EIS}}^{60}>R_{p}{}_{\mathrm{EIS}}^{120}$) in comparison to *R*_p_ measured at *E*_corr_, although the differences between $R_{p}$ values measured by EIS at OCP were rather small. Summarizing, the SMAT process does influence the behavior of magnesium in corrosion, making it more reactive which, in turn, induces the formation of the layer of the oxidation products at the surface, leading to apparent better corrosion resistance. That conclusion is in line with the results of the VEP beam measurements, which showed that the SMATed samples are more susceptible to aerial oxidation and etching. Note that in our experiments, stagnant NaCl solution saturated with Mg(OH)_2_ was used which facilitated the precipitation of magnesium hydroxide at the surface. Such interpretation also agrees with the explanation of the influence of SPD methods on the corrosion resistance of metals given in the paper of Ralston et al. [117]. According to those authors, in the case of corrosion, the real factor influencing the behavior of a metal is the density of grain boundaries at the surface, which influences the activity of the metal in the process of anodic oxidation.

However, our results suggest that not only grain/subgrain boundaries at the surface but also other crystal defects, like dislocations, vacancies, and voids play a significant role in the corrosion behavior. For both the specimen SMATed with 60 s and 120 s, the XRD analysis gave almost the same size of crystallites and only a small difference in the lattice strain (Section 3.2). The lattice strain can be related to all lattice defect density. On the other hand, the crystallite size can be related to the density of the geometrically necessary dislocations (GND) defined as dislocations needed to compensate local misorientation [118]. However, it says nothing about other defects, e.g., statistically stored dislocations (SSDs). They are relevant because one can expect that the density of SSDs should be much higher than the density of GNDs due to a high cumulated plastic strain with some statistical changes in the strain path, which is characteristic for SMAT induced deformation. Nonetheless, the actual dislocation density influences PAS results, which gives clear evidence that the density of structure faults is much higher in the case of samples treated for 120 s than the one treated for 60 s. At the same time, significant differences in the behavior in electrochemical experiments were observed.

It is well known that contamination with attrition media material, especially with Fe, during SMAT can affect the behavior of treated material during electrochemical tests. For example, Jiang et al. reported increased corrosion rate of zirconium due to contamination with Fe and Cr during SMAT [119]. Fabijanic et al. attributed increase in corrosion rate of pure Mg after SMAT to contamination with nanoscale embedded Fe wear debris originating from steel balls, as well as the steel chamber used to perform SMAT [39]. In our case, no iron on the surface of our samples, both before and after the SMAT process, was detected by SEM-EDS analysis.

## 4. Summary

The deformed layer created by SMAT in pure magnesium has been investigated. Polarized light microscopy and EBSD images revealed a gradient microstructure within the deformed surface layer of the SMATed magnesium. A significant decrease in the grain size close to the surface and the gradually decreasing density of characteristic twins belonging to different twinning systems with increasing distance from the treated surface were observed. This result corresponds well with the thickness of the SMAT affected layer determined by microhardness tests and PAS.

The presence of the plastically deformed region rich in crystal lattice defects near the surface was indicated by the high values of the mean positron lifetime equal to 240 ± 1 ps for the samples SMATed for 60 s with the vibration amplitude of 13 ± 2 µm and 244 ± 1 ps for the sample SMATed for 120 s with the vibration amplitude of 27 ± 2 µm. These positron lifetimes, much longer than for the reference sample, point out trapping of positrons in vacancies associated with dislocations and dislocation jogs. The higher energy imparted to the sample surface during longer SMAT with the higher vibration amplitude caused the appearance of a plateau of the high mean positron lifetime values extending to the depth which coincide with the thickness of the highly deformed layer, i.e., about 200 μm. Such a plateau was not present for the shorter SMAT duration with the lower vibration amplitude. The obtained depth profiles of the mean positron lifetime reflect the defect concentration profiles. The decrease of the mean positron lifetime with the depth corresponds with the decreasing density of twins.

The SMAT process strongly influenced the behavior of the magnesium samples during electrochemical corrosion tests. The structure changes induced by SMAT increased the susceptibility of magnesium to anodic oxidation, leading to the enhanced formation of hydroxide coverage at the surface and, as a consequence, leading to apparent better corrosion resistance. This is confirmed by the VEP measurements which indicate a thicker oxide layer on the surface of the sample SMATed for 120 s. Taking into account PAS results, this demonstrates that not only grain/subgrain boundaries at the surface but also other crystal defects, like dislocations, vacancies, and voids, may also play a significant role in corrosion behavior.

## Figures and Tables

**Table 1:** Calculated values of lattice crystallite size for magnesium samples.

Sample	Crystallite Size [nm]
Reference	205 ± 63
SMAT 60 s SMAT 120 s	38 ± 3 37 ± 3
Thickness Reduction 10% Thickness Reduction 40%	48 ± 6 35 ± 5

**Table 2:** Results of fitting of the S(E) curves. L_+layer_ is the positron diffusion length in the surface layer of the samples, which is different from the magnesium matrix, and d is the thickness of this surface.

Sample	L_+layer_ [nm]	d [nm]
SMAT 60 s	74 ± 4	148 ± 9
SMAT 120 s	74 ± 2	370 ± 32
reference	21 ± 10	80 ± 43
SMAT 60 s & etched	6 ± 2	320 ± 2
SMAT 120 s & etched	12 ± 1	708 ± 78
Compressed & Etched	6 ± 1	266 ± 18

**Table 3:** Results of the electrochemical experiments carried out on Mg samples subjected to SMAT duration 0, 60, and 120 s in 0.15 mol dm^−3^ NaCl solution saturated with Mg(OH)_2_ : open circuit potential (OCP) after 2 h, corrosion potential (*E*_corr_), the difference between OCP and *E*_corr_, polarization resistance at the corrosion potential (*R*_p_), corrosion current density (*I*_corr_) obtained by the extrapolation of the cathodic branch of polarization curve in the semi-logarithmic coordinates to *E*_corr_, product of *I*_corr_ and *R*_p_, and polarization resistance measured by electrochemical impedance spectroscopy at OCP.

Time [s]	OCP [V]	*E*_corr_ [V]	*E*_corr_*–*OCP [V]	*R*_p,LSV_ [Ωcm^2^]	*j*_corr_ [Acm^−2^]	*R*_p_*×*jcorr [V]	*R*_p,_EIS [Ωcm^2^]
0	−1.601	−1.528	0.073	173	1.32 × 10^−4^	0.0228	59
60	−1.586	−1.448	0.138	636	5.00 × 10^−5^	0.0318	46
120	−1.576	−1.354	0.222	1612	2.03 × 10^−5^	0.0327	34
